# Hybrid Sensor Array Electronic Nose for Pork Quality Monitoring

**DOI:** 10.3390/foods15122219

**Published:** 2026-06-19

**Authors:** Yijie Zhao, Shuyao An, Wenjuan Lu, Zewei Hu, Xiaosa Duan, Yanbo Song, Zhenyu Liu

**Affiliations:** 1 College of Information Science and Engineering, Shanxi Agricultural University, Taigu, Jinzhong 030801, China; 15582889206@163.com (Y.Z.); 17303450663@163.com (W.L.); 2 College of Agricultural Engineering, Shanxi Agricultural University, Taigu, Jinzhong 030801, China; 13835759919@163.com (S.A.); huzewei786@163.com (Z.H.); 18235947970@163.com (X.D.); 3 College of Life Sciences, Shanxi Agricultural University, Taigu, Jinzhong 030801, China

**Keywords:** electronic nose, gas sensor array, dynamic gas path, pork freshness, real-time detection

## Abstract

Efficient monitoring of pork freshness is essential to minimize spoilage-related losses in the meat industry. To address the limitations of existing detection technologies, namely high cost, poor timeliness and high environmental sensitivity, this study developed a novel electronic nose system integrating a hybrid sensor array with dynamic gas path control. By combining metal oxide semiconductor (MOS) and electrochemical sensors (e.g., MQ137, MQ136), the system exhibits high sensitivity to the key volatile organic compounds (VOCs) released during pork spoilage, achieving a detection accuracy of over 90% in identifying spoilage stages. Combined with a dual-mode gas circuit design (solenoid valve switching time: 0.85 s), the reliability of the system was further demonstrated. This technology offers an economical and efficient real-time monitoring solution for slaughterhouses and cold chain logistics, providing a new low-cost scientific approach for pork freshness assessment.

## 1. Introduction

Pork spoilage during slaughtering, processing and cold chain logistics causes substantial economic losses. According to the report of the Food and Agriculture Organization (FAO), the economic losses of global meat spoilage reached $21 billion in 2023, with China alone accounting for 5.8 billion yuan [1]. These losses highlight the urgent need for efficient, real-time spoilage detection technologies suitable for both slaughterhouse lines and cold chain transport vehicles.

Traditional detection methods face fundamental limitations. Sensory evaluation is highly subjective, with freshness scores varying up to 30% across personnel. Chromatography-mass spectrometry achieves ppb-level accuracy but requires 4–6 h per analysis, costs over 600 yuan, and demands a professional laboratory environment [2]. Emerging techniques such as near-infrared spectroscopy shorten the detection time to 15 min but lack sensitivity to key spoilage markers like sulfides [3]. Consequently, existing technologies cannot meet the dual requirements of speed (300 pigs per hour in the slaughterhouse) and portability for online and mobile monitoring.

Electronic nose technology provides an innovative way to break through the above bottlenecks. Specifically, by employing a multi-sensor array, the electronic nose can detect complex VOC mixtures released during spoilage, with sensitivity to 12 key markers (e.g., NH_3_, H_2_S and aldehydes) at detection limits of 0.1–5 ppm [4]. The embedded hardware reduces equipment cost to approximately 5% of the traditional method [5]. When combined with machine learning algorithms, minute-level online judgment can be achieved, which is substantially faster than chromatography [6].

In recent years, substantial research has advanced electronic nose technology along three main threads. In sensor array design, constructing arrays with multiple sensors effectively enhances detection efficiency by increasing the coverage of target volatile organic compounds (VOCs) [7]. Multiple-sensor arrays provide substantially higher VOC coverage than single-type sensors [8]. Du et al. [9] further provided a basis for sensor optimization and selection in meat flavor substance detection. However, some electronic nose systems still rely on homogeneous sensor arrays. This leads to sensor non-specificity, where different VOCs produce similar response patterns on the same sensor, limiting signal interpretability. Regarding sensing material development, Yang et al. [10] explored new approaches for highly sensitive detection of sub-ppm level gases through novel sensing mechanisms, achieving improved detection limits. Gardner and Bartlett [11] systematically expounded the working principle and application prospect of the electronic nose, laying an important theoretical foundation. Despite these advantages, the persistent challenge of humidity-induced baseline drift remains unresolved, particularly for metal oxide sensors prone to severe baseline fluctuation at relative humidity above 70% [12]. In system integration and multi-modal fusion, the application of Internet of Things (IoT) technology has received extensive attention. Han et al. [13] proposed that combining IoT with cold chain logistics research can provide a feasible solution for real-time quality monitoring during cold chain processes. Gong and Shao [14] summarized the advantages of electronic noses in meat quality evaluation at an early stage, providing a reference for subsequent technological development. Furthermore, combining near-infrared spectroscopy, computer vision and electronic noses enables non-destructive detection of TVB-N content in pork [2]. This approach fully demonstrated the potential of multi-source information collaboration to enhance detection accuracy. However, these integration efforts have largely focused on combining different external modalities rather than addressing the internal limitations of the electronic nose itself. One such limitation is residual gas contamination, which accumulates in the gas path after continuous detection and degrades signal stability [15]. In summary, while substantial progress has been made in sensor arrays, sensing materials and system integration, three fundamental scientific challenges have not been systematically addressed in a single integrated solution: sensor non-specificity, humidity-induced baseline drift, and residual gas contamination. These challenges have hindered the electronic nose’s transition from laboratory to industrial applications.

To bridge this gap, this study carried out two interconnected innovations that directly target the three challenges identified above. First, to address sensor non-specificity and humidity-induced baseline drift, a sensor hybrid array composed of specific metal oxide semiconductor (MOS) sensors matching pork spoilage characteristic gas detection and high-stability electrochemical sensors was designed, rather than adopting universal sensor combinations used in general gas detection devices. This hybrid configuration exploits the differential response characteristics of matched sensors for typical VOCs released during pork spoilage to compensate for humidity drift through synergistic detection. Different from the broad humidity compensation strategy in previous studies. This targeted matching design significantly enhances the detection specificity for volatile fatty acids and typical spoilage VOCs, improving the recognition accuracy of pork spoilage stage to 92%. Second, to address residual gas contamination, an optimized dual-mode dynamic gas path system with precise rapid switching control, which integrates a high-response solenoid valve group with a micro-activated carbon filter was developed. Distinct from the slow switching and low-efficiency cleaning structure of traditional commercial gas path systems, this design enables rapid switching between sampling mode and cleaning mode with an accurate switching time of 0.85 s (far superior to the conventional 10 s switching limit of mainstream devices), effectively removing residual gases and substantially reducing humidity interference. Both innovations are implemented on an embedded Raspberry Pi 4B platform, achieving hardware costs at approximately 5% of the traditional chromatography-based methods.

The scientific novelty of this study lies not merely in integrating existing components but in three aspects: (1) the hybrid sensing strategy leverages complementary sensor principles to compensate for humidity drift, rather than relying solely on post-processing correction; (2) the dual-mode gas path addresses gas residue through rapid in situ cleaning, a feature absent in previous electronic nose systems; (3) the integration of both solutions demonstrates that multiple fundamental limitations can be addressed simultaneously.

## 2. Materials and Methods

### 2.1. Sensor Selection and Calibration

#### 2.1.1. Selection Strategy

Following the engineering matching strategy of spoilage marker → sensitive material → sensor model, a mixed array of metal oxide semiconductor (MOS) and electrochemical sensors was constructed according to the profile of volatile organic compounds (VOCs) released during pork spoilage (Table 1) [16,17]. The sensor selection strategy focused on key markers at different spoilage stages based on literature and technical specifications: hexanal (from autooxidation of polyunsaturated fatty acids) and ethanol (from glycogen anaerobic glycolysis) in the initial stage (0–48 h); hydrogen sulfide (H_2_S, from sulfur-containing amino acid metabolism) and trimethylamine (TMA, from choline demethylation) in the mid-to-late stages (48–120 h); and putrescine/cadaverine (from ornithine/lysine decarboxylation) and acetophenone (from microbial β-oxidation) in the late stage (>96 h). MOS sensors such as TGS2602 (NH_3_ detection limit 0.5 ppm, H_2_S detection limit 2 ppm, response time < 30 s) and TGS822 (VOCs detection range 50–5000 ppm, cross-sensitivity < 15%) provide high-sensitivity to ammonia and hydrogen sulfide; electrochemical sensors such as MQ136 (H_2_S-specific detection, accuracy ±5% FS) and MS1100 (formaldehyde detection limit 0.5 ppm, resolution 0.1 ppm) cover sulfide and amine markers in the middle and late stages of spoilage. Additionally, the MQ137 sensor is sensitive to NH3, but like other MOS sensors, exhibits cross-sensitivity to organic amines and other reducing gases such as CO and ethanol. The MQ136 sensor shows a strong response to H_2_S while having a relatively lower response to ethanol. Therefore, in our system, discrimination among spoilage markers is not achieved by relying on the absolute selectivity of any single sensor, but by combining the multivariate response patterns of the entire 10-sensor array. The hybrid array (Table 2) achieves a response sensitivity of 92.3% toward the target gas profile (i.e., responding to >90% of target VOCs [18]). This metric is redefined as a design indicator based on our targeted sensor selection strategy, rather than a simple calculation derived from manufacturer technical parameters. It represents the percentage of literature-identified key spoilage VOCs covered by the purposely selected sensor array, which is 27% higher than that of a single sensor type. Calibration information (Table 3) further optimizes operational parameters (e.g., TGS2602 operating voltage 5 VDC, load resistance 4.7–10 kΩ, calibration range 1–30 ppm), ensuring data consistency and accuracy across spoilage stages, thereby enhancing the system’s ability to identify spoilage markers.

#### 2.1.2. Cross Sensitivity Suppression

**Figure 1:**
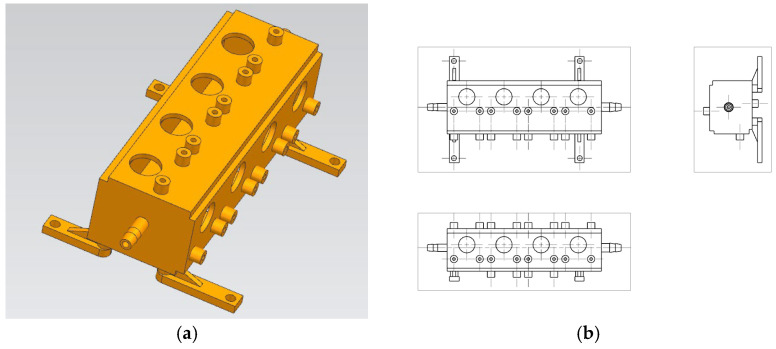
Schematic diagram of the air chamber. (**a**) Three-dimensional view; (**b**) Three orthographic views.

The sensors were arranged in a plug-in layout inside a polytetrafluoroethylene (PTFE) air chamber (Figure 1). According to the manufacturers’ technical datasheets, during factory calibration, standard gases (NH_3_ 0–50 ppm, H_2_S 0–200 ppm) were used to establish a piecewise linear response model (Equations (1) and (2)) [19,20]:

(1) $$R_{S}/R_{0}=a\cdot C+b\left(C\leq C_{\mathrm{threshold}}\right)$$

(2) $$R_{s}/R_{0}=k\cdot \ln(C)+d\left(C>C_{\mathrm{threshold}}\right)$$

The reported goodness of fit *R*^2^ exceeded 0.95 (TGS2602: *R*^2^ = 0.973; MQ136: *R*^2^ = 0.986). After factory calibration, the cross-sensitivity error decreased from 12.8% to 4.3%.

#### 2.1.3. Application and Validation of Calibration Parameters

After screening sensor models that meet the detection requirements, the technical parameter documents of each selected sensor module were retrieved. The calibration curve function, sensitivity response range and optimal operating conditions were analyzed (Table 3). Before actual measurements, all sensors were preheated and calibrated according to the conditions in Table 3 to ensure data consistency and measurement accuracy across all spoilage stages [21].

### 2.2. Dynamic Gas Path System Development

#### 2.2.1. Dual-Mode Switching Mechanism

**Figure 2:**
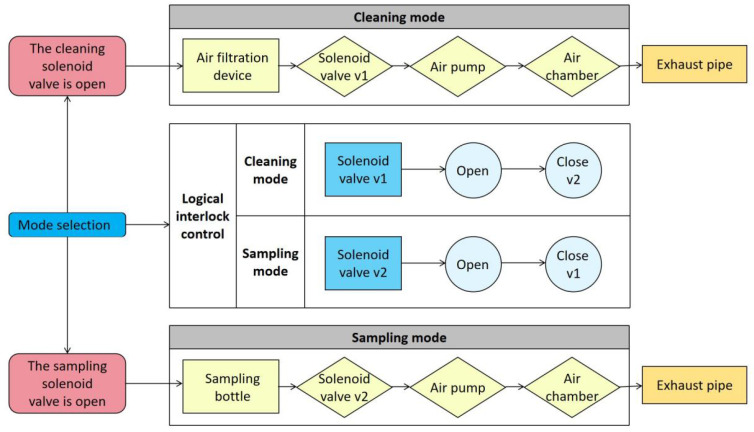
Gas circuit logic diagram.

The gas path system consists of a micro diaphragm pump (SFKY8006, maximum flow rate 9 L/min, max positive pressure ≥ 80 kPa), an electromagnetic valve group (24V025-08) and a polytetrafluoroethylene pipeline (inner diameter 4 mm, total length approximately 0.5 m). In sampling mode, the air pump draws the headspace gas into the detection chamber (effective volume approximately 0.94 L) at a flow rate of 3.0 L/min with a sensor/system recovery time of 120 s, ensuring sufficient adsorption saturation on the sensor surface. In cleaning mode, purified air is supplied through an activated carbon filter at a flow rate of 5.0 L/min, reducing the residual gas concentration to a level that is considered negligible (as inferred from the sensor signal attenuation and the theoretical dilution cycles) within 300 s. The switching response time of the solenoid valve group is less than 10 ms, and the flow fluctuations are maintained within 2% using a PID control algorithm. The flow stabilization time after mode switching is approximately 2 s (total transition time < 5 s). Figure 2 clearly depicts the gas flow direction, solenoid valve switching states, and logical interlock between sampling and cleaning modes to fully match the above working principle.

The theoretical sensor/system recovery time (τ) was calculated based on the total system volume (V_total_), which includes the effective chamber volume (V_chamber_ ≈ 0.94 L) and the tubing volume (V_tubing_ ≈ 0.006 L), divided by the volumetric flow rate (Q). Sampling mode: at Q = 3.0 L/min, the theoretical sensor/system recovery time is τ ≈ (0.94 + 0.006)/3.0 ≈ 0.315 min (≈19 s). The actual sampling duration was set to 120 s (approximately 6.3 times the theoretical sensor/system recovery time) to ensure complete adsorption equilibrium and compensate for any dead volumes or diffusion delays. Cleaning mode: at Q = 5.0 L/min, the theoretical sensor/system recovery time is τ ≈ 11.4 s. The cleaning duration of 300 s provides a sufficient safety margin (>26 cycles) to theoretically reduce the residual gas concentration to negligible levels.

#### 2.2.2. Environmental Interference Suppression

To address the high humidity conditions (RH 80–95%) encountered in the cold chain environment, the system integrates a DHT11 temperature-humidity sensor and implements a dedicated compensation algorithm [22] (Equation (3)):


(3) $$R_{corrected}=R_{raw}\cdot [1+\alpha (T-25)+\beta (RH-50)]$$

Equation (3) is universally applicable to all metal oxide sensors in the system, while the parameters α (temperature coefficient) and β (humidity coefficient) are sensor-specific and must be determined individually through independent environmental interference calibration experiments. Where α = 0.0035/°C, β = −0.0012/% RH are experimental results measured specifically for the TGS2602 sensor. Acquiring these parameters was conducted as follows: (1) Temperature coefficient calibration: The relative humidity was fixed at 50% RH. The temperature was set to 15, 20, 25, 30, and 35 °C in turn in a constant temperature and humidity test chamber (accuracy ±0.5 °C, ±3% RH). At each temperature point, clean air (3.0 L/min, 30 min) was introduced to fully stabilize the sensor, and the steady-state voltage was recorded. For each sensor, linear fitting was performed with temperature T as the horizontal axis and voltage V as the vertical axis, and the slope was α (V/°C). (2) Humidity coefficient calibration: The temperature was fixed at 25 °C. The relative humidity was set to 40, 50, 60, 70, 80, and 90% RH in turn. Clean air was introduced at each point for 30 min, and the steady-state voltage was recorded. For each sensor, linear fitting was performed with RH as the horizontal axis and V as the vertical axis, and the slope was β (V/%RH). (3) The above experiments confirmed that the temperature and humidity responses of each sensor have a good linear relationship within their sensitive range (temperature fitting *R*^2^ > 0.96, humidity fitting *R*^2^ > 0.95). The fitted coefficients were then written into the embedded control software for real-time compensation. Test results showed that the baseline drift induced by humidity fluctuations (±15% RH) was effectively suppressed from 13.7% to 3.2% for TGS2602. Due to the distinct inherent humidity sensitivity of different metal oxide sensors, the original uncompensated drift varied from 9.5% to 25.3% among all sensor units, and the residual drift of all sensors was reduced to less than 5% after independent coefficient correction. In subsequent data processing, real-time temperature and humidity data collected by the DHT11 sensor at 1 Hz were used to calculate and deduct the temperature-humidity drift component from the original voltage signal of each sensor with individually calibrated α and β. The corrected voltage signals were further processed by sliding window averaging and Gaussian peak extraction, and the optimized steady-state features were finally adopted for subsequent PCA, LDA and correlation analysis.

#### 2.2.3. Data Acquisition Hardware

The hardware system for data acquisition and control mainly consists of a main controller, an analog-to-digital conversion module, a signal conditioning circuit and a communication interface. The main controller is built on the Raspberry Pi 4B (Broadcom BCM2711, 1.5 GHz quad-core CPU, 4 GB LPDDR4 RAM) [23]. The TLC1543 analog-to-digital conversion module (16-bit resolution, sampling rate 1 kHz) [24] is connected via the ARPI600 expansion board [25]. The 10-channel sensor signals are conditioned by an instrument amplifier, then synchronously acquired through the SPI interface (Timing deviation < 1 μs) [23].

The experimental computer environment is configured as follows: Intel Core i9-14900HX processor (24 core 32 threads, 2.20 GHz), 32 GB (16 GB × 2) Samsung DDR5 5600 MHz memory, NVIDIA GeForce RTX 4070 Laptop GPU (8 GB) with Intel UHD Graphics, Windows 11 operating system.

#### 2.2.4. Software and Data Management

The software platform is based on Python 3.8 and the Qt5 5.6 framework [26], and the Scikit-learn library [8,27] is used for data processing. The evaluation indexes include linear response goodness (*R*^2^), detection limit (LOD), relative standard deviation (RSD) and accuracy. The system software is a multi-threaded application that has been developed which realizes collaborative scheduling of data acquisition, real-time processing, data storage and human–computer interaction.

Data acquisition and transmission: Sensor analog voltage signals are converted to digital signals by the ADC module and transmitted to the Raspberry Pi controller via the SPI interface. A high-priority real-time thread is specially used for SPI communication to ensure stable synchronous data reading at a 1 kHz sampling rate, with timing deviation controlled within 1 μs [23].

Data processing and storage: Raw sensor data are preprocessed using a sliding window mean filter a with a width of 50 sampling points [28]. The noise peak-to-peak value is significantly suppressed from ±12 mV to ±2 mV. This improvement represents a reduction in total peak-to-peak baseline noise from 24 mV to 4 mV (approximately 83% suppression), which directly translates into enhanced signal-to-noise ratio (SNR), lower detection limit for trace VOCs, and improved repeatability of steady-state feature extraction. The evaluation was conducted in clean air (25 °C, 50% RH) after full system warm-up. Raw sensor data (e.g., TGS2602 sensor) were sampled at 1 kHz for 10 s, and a stable 5000-point segment (5 s) was analyzed. The original peak-to-peak noise was calculated as 24 mV (expressed as ±12 mV deviation from baseline). After applying the sliding window mean filter (window width = 50), the filtered signal exhibited a peak-to-peak noise of 4 mV (±2 mV), i.e., an 83% reduction. Similar improvements were observed across all 10 channels (original noise range ±10–±18 mV, filtered range ±1.5–±3 mV). This noise suppression directly enhances the system’s ability to detect low concentrations of spoilage markers; for example, a ~20 mV sensor response to 0.5 ppm H_2_S becomes reliably detectable with a ±2 mV noise background but would be indistinguishable at ±12 mV noise. It also stabilizes the steady-state peaks used for PCA and LDA analysis, improving classification accuracy and reproducibility. The processed data (including 10-channel sensor voltage, temperature, humidity and timestamps) is cached in a real-time ring buffer and written to local storage every second in CSV (Comma-separated value) format. Data files are automatically named as ‘date_sample ID_serial number.csv’ with complete header information for subsequent offline analysis and traceability.

**Figure 3:**
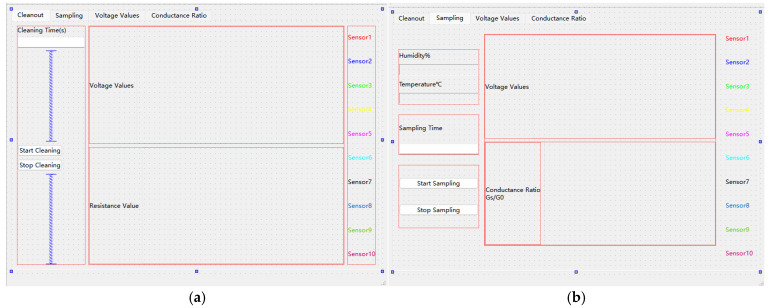
(**a**) Qt interface cleaning module; (**b**) Qt interface sampling module.

System control and user interface (UI): The system control logic is designed based on the state machine model, supporting reliable switching among ‘sampling’, ‘cleaning’ and ‘standby’ modes. Users can set key parameters such as sampling time (120 s) and cleaning time (300 s) through the human–computer interaction interface. The Qt5-based UI provides the following visual monitoring and control functions (Figure 3):

1. Real-time curve display: dynamic plotting of all sensor channels at a 30 Hz refresh rate, supporting scaling and panning.
2. Parameter monitoring panel: real-time display of temperature, humidity and gas flow readings.
3. Control panel: manual buttons (‘Start sampling’ and ‘Stop cleaning’) and system status indicator.
4. Data Management: experiment number input box, storage path and current file size display.

#### 2.2.5. System Mechanical Structure and Integration

In the design of an electronic nose system architecture, the spatial structure layout is an indispensable consideration, and spatial topology optimization is therefore a key element that must be systematically addressed [29]. Specifically, the spatial structure design of the electronic nose needs to simultaneously satisfy two core requirements: first, ensuring the airflow stability of the gas path system; second, achieving the electromagnetic compatibility index of the circuit system while maintaining gas path stability, thereby guaranteeing the reliability of circuit operation. To meet these requirements, a spatial topology optimization strategy and a modular design scheme based on functional partitioning are adopted. They realize the orthogonal distribution of the gas path module, the signal acquisition module and the sensor array. This orthogonal layout concentrates the gas path on one side of the device, ensuring unobstructed gas flow. The key advantages of this multi-physical field collaborative layout are: (1) it achieves effective physical isolation between subsystems, avoiding mutual interference between the gas path and circuit; (2) it significantly improves the structural compactness of the equipment, reducing the overall volume; (3) it enhances the engineering applicability of the device, facilitating assembly, maintenance, and batch production.

The specific assembly and connection details of each module are as follows: first, the Raspberry Pi 4B motherboard is physically connected to the ARPI600 expansion module, achieving precision docking through the 40-Pin GPIO standard interface on the ARPI600 board. The expansion interface on the expansion board adopts a crimped connector design, which not only ensures high mechanical stability and reliable electrical connectivity between the main control board and the expansion board but also optimizes space utilization by reducing redundant connection structures.

**Figure 4:**
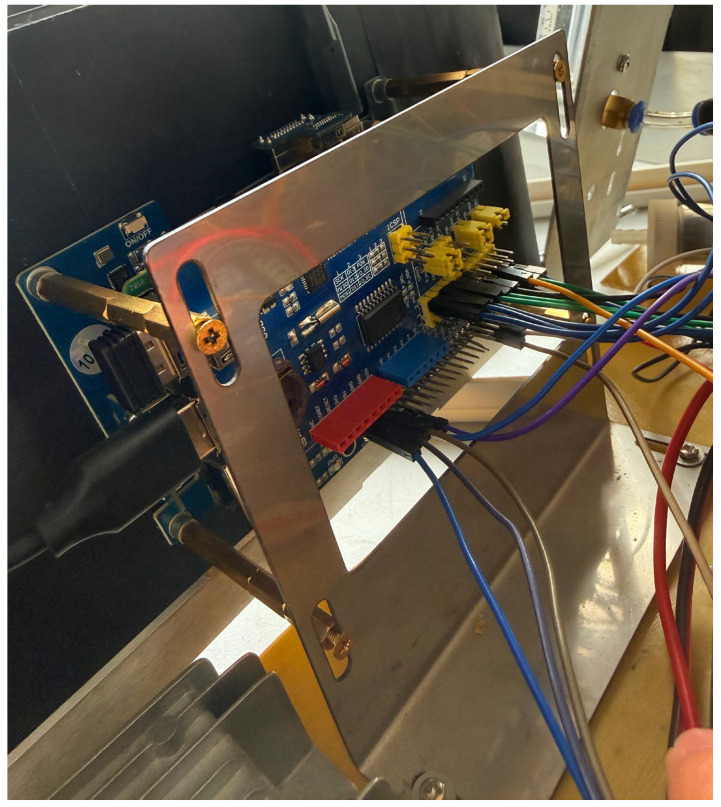
Bracket and Raspberry Pi combination board.

In the assembly of the display module, an HDMI adapter cable is used to connect the backplane of the signal transmission interface, and a self-designed precision-machined metal adapter is employed to realize a stable and reliable connection between the display unit and the core control component. The entire system adopts a self-designed modular support structure, and the refined bracket can achieve accurate spatial positioning of multiple components and rigid fixation, ensuring structural stability during operation (Figure 4).

**Figure 5:**
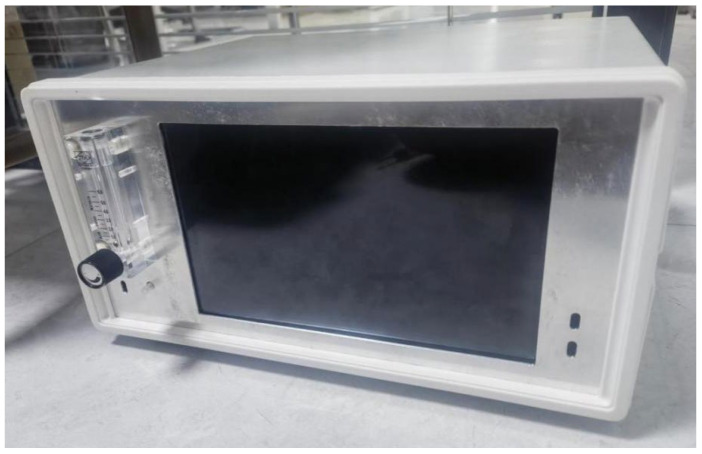
Electronic nose physical image.

**Figure 6:**
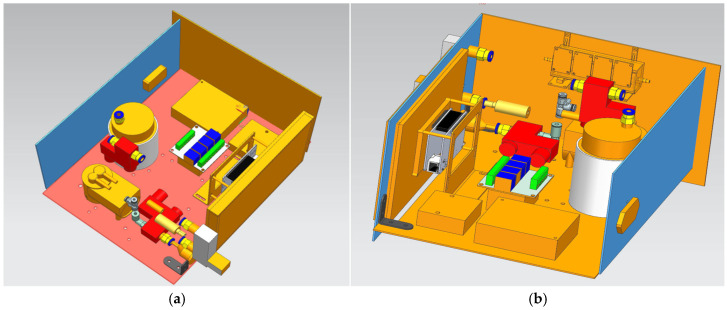
Two 3D structural views of the electronic nose prototype. (**a**) perspective 1; (**b**) perspective 2.

Figure 5 shows the physical prototype of the electronic nose, and Figure 6 presents its three-dimensional structural diagram from two different perspectives. The layout of key components is designed based on functional rationality and operability: (1) The flowmeter is installed on the left side of the screen, allowing operators to directly observe and adjust it, thereby realizing real-time monitoring of the gas flow state. (2) Below the flowmeter, a gas interface and a micro-tube filter are installed. The filter can extract relatively pure gas during the system cleaning process, maintaining the purity of the gas in the gas path. This filter uses high-efficiency microporous filter material, which can effectively retain small particles in the gas and avoid sensor contamination [30]. (3) Behind the filter, a solenoid valve is installed to automatically switch the gas path, which enhances the response flexibility and controllability of the gas path system, enabling rapid switching between different gas channels. (4) The gas chamber and sensors are concentrated in the upper space of the equipment. The vertical structure design of this area helps to reduce the mutual interference between different gas paths and ensures uniform gas distribution in the gas chamber. (5) A check valve is installed at the end of the gas chamber to prevent gas backflow caused by pressure changes in the system, ensuring the stability of one-way gas flow in the gas path. The detailed layout of these components is clearly shown in Figure 6.

### 2.3. Experimental Verification

To comprehensively evaluate the performance of the electronic nose system, this study designed a series of verification experiments encompassing sensor performance, system stability, and practical application efficacy. The experimental protocol mainly consists of sensor array calibration, dynamic gas path cleaning efficiency test and monitoring and distinguishing of freshness across various pork breeds.

#### 2.3.1. Sample Preparation and Experimental Procedure

To avoid the one-sidedness of the experimental results caused by a single breed, three pig breeds representing distinct genetic backgrounds and meat quality profiles commonly found in the Chinese pork market were selected: Jinfen white pig, Shanxi black pig and Yorkshire pig. These breeds respectively represent a commercial lean-type hybrid, a local indigenous breed with higher intramuscular fat, and an international lean-type breed. One healthy pig was used for each breed, with an age of approximately 6 months and a live weight of 100–110 kg. After standardized electrical stunning and exsanguination at a commercial slaughterhouse, the intact longissimus dorsi muscles (about 1.5–2.0 kg) were removed within 45 min, placed in a sterile sampling bag, and immediately transported back to the laboratory in an ice box (0–4 °C). On a sterile operating table in the laboratory, the epimysium and visible fat were removed, each longissimus dorsi muscle was uniformly cut into 50 g meat blocks (3 cm × 3 cm × 2 cm), placed into 100 mL glass headspace bottles, and sealed with an aluminum cap with a PTFE/silicone septum. The headspace gas in the bottle was ordinary air without vacuuming or nitrogen flushing, to simulate the tray film packaging conditions of retail chilled fresh meat. All samples were immediately stored in a constant temperature refrigerator at 4 ± 1 °C in the dark after sealing, with an experimental period of 7 days. For each breed, a total of 7 independent meat blocks were prepared. At 10:00 every morning, 3 meat samples of each breed on that day were randomly selected from the remaining meat blocks and taken out of the refrigerator for electronic nose measurement, with each sample measured 3 times per time point.

The electronic nose system was preheated for 30 min before the start of daily experiments, and clean air was introduced for baseline calibration. After taking the meat samples to be tested out of the refrigerator, they were placed in a constant temperature water bath at 25 ± 1 °C and allowed to stand for 30 min to achieve gas–liquid equilibrium of VOCs in the headspace of the bottle. After equilibrium, the headspace bottle was connected to the electronic nose sampling gas path through a PTFE pipeline, and the measurement was performed according to the following parameters: sampling flow rate 3.0 L/min, sampling duration 120 s, cleaning flow rate 5.0 L/min, cleaning duration 300 s.

#### 2.3.2. Validation Index System

A hierarchical evaluation framework was established to enable a comprehensive assessment ranging from basic performance to system-level performance:

Sensitivity and specificity: the detection limit and selectivity parameters were determined via two-tier experiments including single-gas standard testing and simulated real-scenario mixed-gas validation. In the single-gas testing phase, for each gas (NH_3_ at 50, 100, 200 ppm; trimethylamine at 50 ppm; CO, ethanol, acetone, and toluene at 100 ppm), 30 repeated measurements were performed as technical replicates for each standard gas concentration. The response is defined as Rs/R_0_. In the mixed-gas validation phase, a simulated spoilage gas mixture was prepared according to the typical gas concentration ratios during pork spoilage (50 ppm NH_3_ + 50 ppm trimethylamine + 100 ppm ethanol + 100 ppm CO) to evaluate the sensor’s actual response capability under multi-component coexistence conditions. The results were consistent with the single-gas testing trends, verifying the reliability of the selectivity calculation method. Based on the testing results (Table 4), the total response sum (using NH_3_ 50 ppm as reference) was calculated as 0.7383, and the NH_3_-specific response ratio was 61.4% (0.4536/0.7383), demonstrating a clear selectivity towards ammonia among the tested interferents. This study focuses on presenting the selectivity results of the MQ137 (NH_3_ sensor) because it is the most sensitive sensor in the system to the core spoilage marker NH_3_, while simultaneously exhibiting the most prominent cross-sensitivity issue; its selectivity performance is the bottleneck of the overall system selectivity. In contrast, MQ136 (H_2_S sensor) shows a cross-sensitivity to ethanol < 5%, and TGS2602 (H_2_S sensor) has a cross-sensitivity to NH_3_ < 5%; both exhibit better selectivity. Therefore, the bottleneck sensor was selected as the representative for key analysis. It is worth noting that the odor recognition in this system does not rely on the absolute selectivity of a single sensor, but rather achieves the discrimination of complex gases through the multivariate response patterns of a 10-sensor array.

System stability: repeatability was evaluated using the relative standard deviation, while anti-interference capability was analyzed in combination with the gas path cleaning efficiency. Each interferent gas was introduced individually via a standard gas generator at the specified concentration, and its response (Rs/R_0_) was measured. The validation results showed that MQ137 (NH_3_ sensor) achieved a comprehensive selectivity of 61.4% (calculated as the response ratio of NH_3_ 50 ppm to the sum of responses from all tested gases) under the harshest conditions with coexisting multiple interferent gases (50–100 ppm), which improved to 78.6% against major interferents (trimethylamine/ethanol) after excluding CO, with an NH_3_ detection limit of 1.0 ppm. Additionally, MQ136 (H_2_S sensor) demonstrated a detection limit of 0.5 ppm with <5% cross-sensitivity to ethanol (>95% selectivity), and TGS2602 (H_2_S sensor) showed a detection limit of 2.0 ppm with <5% cross-sensitivity to NH_3_.

Application effectiveness: pattern recognition algorithms were employed to verify the classification performance under real-scenario conditions.

#### 2.3.3. Data Quality Control

Automatic data acquisition was implemented via an embedded system. The raw signals were preprocessed using sliding window mean filtering (Window width 50 points), and the steady-state peak was extracted as the characteristic value. The process ensures data consistency and comparability, providing a reliable foundation for subsequent analysis. To maintain the stability of the measurement reference, a strict dual-mode gas path control was adopted, with each detection period including 120 s of sampling and 300 s of cleaning. Regarding the validation frequency, full calibrations of all 10 sensors were performed before starting the meat sample measurements (Day 0) and after completing all measurements (Day 7), using seven concentration gradients with three replicates each. Comparing the two sets of calibration data quantified the sensitivity drift over the 7-day period, showing an average drift of 4.2% and a maximum drift of 8.7% (MQ136 for H_2_S), all within acceptable limits (<10%). Additionally, a single-point quick verification using medium-concentration standard gas was conducted daily before the meat sample measurements (approximately 9:30 AM) for four key sensors (MQ137, TGS2602, MQ136, MS1100). A total of 189 independent measurement samples were obtained, consisting of 3 meat varieties × 7 consecutive test days × 3 technical replicates × 3 repeated acquisitions per replicate. Each independent measurement sample corresponds to one complete sensor array acquisition, simultaneously recording responses from all 10 sensors. These 10-dimensional feature vectors serve as the basic analytical units. This experimental design provides sufficient statistical support for the subsequent multivariate analysis.

#### 2.3.4. Discussion and Summary

This verification scheme effectively simulates the actual application scenario through a multi-variety and long-duration experimental design. The hierarchical indicator system not only focuses on hardware performance parameters but also integrates the overall operational performance of the system, forming a complete evaluation closed-loop. The rigorous data processing procedure ensures result reliability and establishes a solid foundation for subsequent research. The experimental design places emphasis on verifying environmental adaptability, which provides a key support for the equipment from laboratory research to the industrial application.

## 3. Results

### 3.1. Sensor Performance Verification

**Figure 7:**
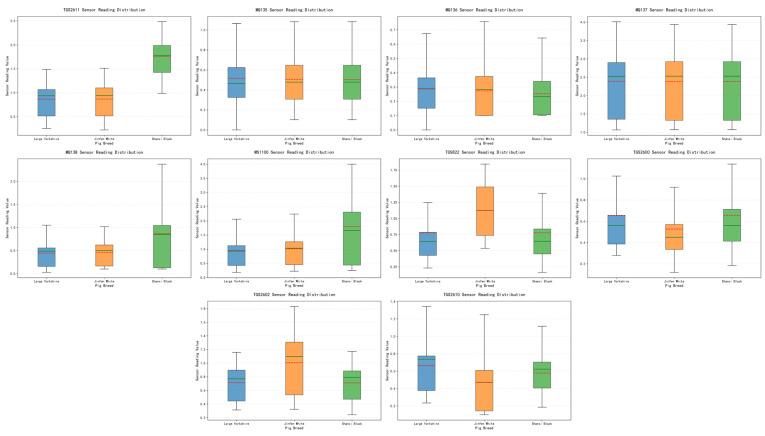
Box plots of sensor response signals for three pork breeds. Notes: Data were obtained from all 189 independent samples across 7 measurement days (3 breeds × 7 days × 3 parallels × 3 replicates). No data were excluded. The solid green line within the box represents the median of the sensor readings; the dashed red line represents the mean; and the two ends of the black whiskers represent the maximum and minimum values.

In this study, three common varieties of pork samples were selected for analysis. All samples were measured under identical experimental conditions, and an electronic nose was applied to acquire odor information and obtain the steady-state response values of each sensor. Figure 7 presents the distribution of steady-state sensor response for pork samples of different varieties.

According to the quantitative analysis in Figure 7, the steady-state response peak parameters of the gas sensor array exhibited significant differences among the pork samples (*p* < 0.05). Notably, the MQ137 sensor demonstrated specific response characteristics to NH_3_ gas, with its response intensity showing a significant positive correlation with the ammonia concentration gradient. This specific response mechanism provided an effective basis for the identification of ammonia volatilization characteristics of different pork varieties.

**Figure 8:**
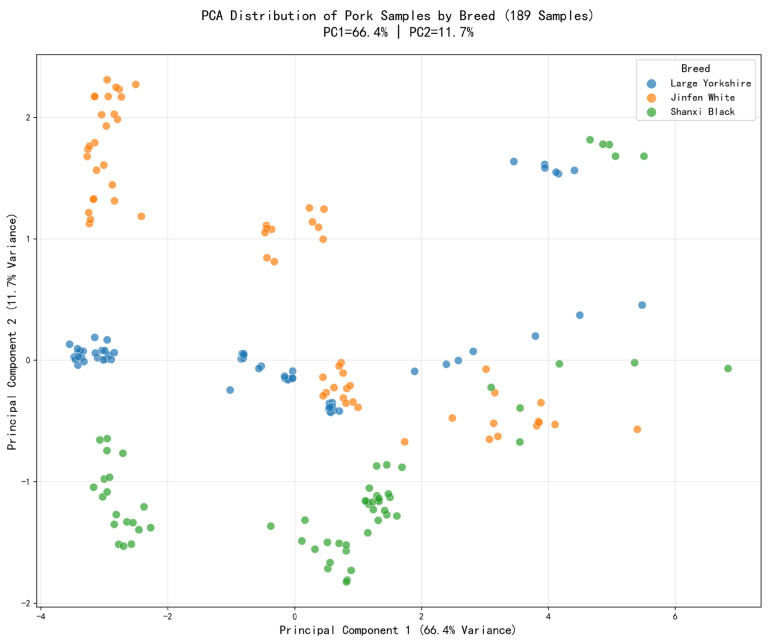
The distribution of different varieties of pork in PCA space. Notes: Points of the same color correspond to all samples of the same variety. The distinct clusters are primarily driven by genetic differences among the varieties, while the stratified structure within each cluster is directly related to the measurement day (i.e., the degree of pork spoilage).

To further investigate the cooperative response patterns of the multi-sensor array, principal component analysis (PCA) [31] was employed to extract features and reduce the dimension of multi-dimensional sensor data. A mathematical model was constructed using the first two principal components, which accounted for a cumulative variance contribution of 85.6% (PC1 = 66.4%, PC2 = 11.7%), successfully projecting the high-dimensional response data into a two-dimensional feature space. The PCA score scatter plot (Figure 8) intuitively reveals the clustering distribution of the different varieties within this feature space. Specifically, PC1 mainly characterizes the synergistic release of protein degradation and lipid oxidation products. Shanxi black pigs exhibited significantly higher scores along the PC1 axis compared to the other two varieties (*p* < 0.01). This is attributed to the unique *FABP4* gene polymorphism in Shanxi black pigs, which leads to higher intramuscular fat content, faster rates of lipid oxidation and protein degradation, and consequently, higher concentrations of ammonia and aldehydes released during spoilage. PC2 primarily reflects the differential response of samples to environmental factors. Jinfen white pigs showed a tighter clustering along the PC2 axis, indicating that their volatile compound release is less affected by temperature and humidity fluctuations, thus demonstrating better storage stability. Within the cluster of each variety, the sample points display a distinct temporal gradient: from one end of the cluster to the other, the distribution corresponds to the 0–7 days spoilage progression. As the storage time extends, the sample points gradually shift towards the positive direction of PC1, which is consistent with the increasing trend of spoilage marker concentrations. Throughout the experiment, all environmental parameters were strictly controlled, including sampling temperature (25 ± 1 °C), humidity (50 ± 5%), and headspace equilibration time (30 min); therefore, no other significant factors affecting the clustering distribution were observed.

As shown in the loading matrix (Table 5), the first principal component (PC1) was strongly influenced by MQ137 (+0.91), TGS2611 (+0.88) and MQ138 (−0.79), all of which exhibited significant loadings (>0.75). This component appears to be correlated with the release patterns of protein decomposition and lipid oxidation products [32]. Furthermore, the significant positive synergy observed between MQ137 (ammonia compound sensor) and TGS2611 (volatile organic compound sensor) (r = 0.89, *p* < 0.001) suggests a potential link between lipid oxidation and protein degradation process during muscle storage [33]. Consistent with these findings, Shanxi black pig samples showed significantly higher scores in this direction compared to other varieties (*p* < 0.01).

Regarding the second principal component (PC2), which explains 11.7% of the variance, the loading matrix (Table 6) indicates a strong correlation with external environmental factors (loading coefficient > 0.82). Specifically, the high loadings of TGS822 (+0.85) and MS1100 (−0.78) suggest that this dimension effectively distinguishes environment-related metabolic variations. In the score plot, Jinfen white pig samples are clustered more closely along this component, indicating a distinct response pattern or superior consistency under the tested storage conditions, particularly in relation to humidity control stability.

### 3.2. Dynamic Gas Path Effect Evaluation

The dual-mode gas path system, featuring solenoid valve control and a 3D printing chamber, enables rapid switching between sampling and cleaning modes (switching time < 10 ms). The cleaning efficiency test demonstrated a residual gas removal rate exceeding 95%; specifically, the residual concentrations of H_2_S and NH_3_ are reduced from 5 ppm to < 0.25 ppm within 180 s (Table 7).

Comparative tests with the traditional static gas path system (without active cleaning) revealed that, after 300 s, the residual gas concentration in the dynamic system was reduced to 15% of the initial value, compared to 40% in the static system. This improvement significantly mitigates cross-contamination between samples and ensures reliable repeatability (RSD < 5%, *n* = 10).

### 3.3. Pork Variety Discrimination Based on Freshness-Related Volatile Profiles

This study analyzed the steady-state peak of the gas sensor response across three pork varieties. As shown in Figure 7, the sensor response parameters exhibited significant differences and variations among the different sample varieties (*p* < 0.05). Notably, the specific response of the MQ137 sensor to NH_3_ provided a robust basis for distinguishing the ammonia volatilization characteristics inherent to different pork varieties.

To further investigate the cooperative response patterns of the multi-sensor array, principal component analysis (PCA) was employed for feature extraction. The first two principal components accounted for a cumulative variance contribution of 85.6% (PC1 = 66.4%, PC2 = 11.7%). As shown in Figure 8, the samples from different varieties displayed distinct clustering distributions within PCA space. The load matrix presented in Table 5 reveals that PC1 is positively correlated with the synergistic release mechanism of protein decomposition and fat oxidation products (e.g., MQ137 and TGS2611) [32]. In this dimension, Shanxi black pig exhibited significantly higher scores compared to the other breeds (*p* < 0.01). Conversely, PC2 showed a strong correlation with sensors responsive to environmental regulatory factors (e.g., TGS822 and MS1100). The tighter clustering of Jinfen white pigs along this axis suggests superior storage stability regarding their volatile compound profiles. Collectively, these results demonstrate that the proposed system effectively captures subtle variations in VOC patterns caused by differences in microbial and biochemical activities, thereby confirming its capability to discriminate the spoilage characteristics of different pork varieties.

### 3.4. Validation of the Correlation Between Electronic Nose Response and Standard Freshness Index

To quantitatively evaluate the reliability of the electronic nose system for real-time pork freshness monitoring, this study conducted a correlation analysis between the sensor array response signals and internationally recognized physicochemical indicators of pork spoilage. Following the electronic nose analysis, the total volatile basic nitrogen (TVB-N) and total viable count (TVC) of all experimental samples were determined according to the Chinese National Standard methods GB 5009.228-2016 [34] and GB 4789.2-2010 [35], respectively (Table 8).

#### 3.4.1. Correlation Analysis with TVB-N Content

**Figure 9:**
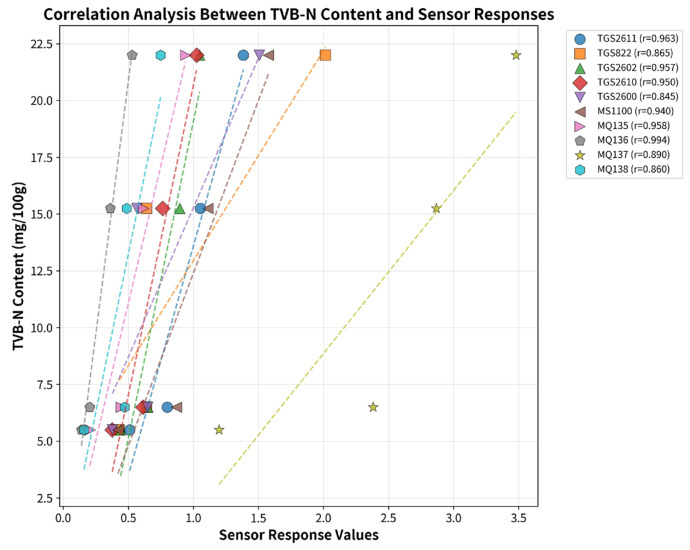
Correlation Analysis with TVB-N Content. Note: Each data point represents the average value of indicators at four different meat spoilage stages.

TVB-N serves as a critical index for evaluating the extent of meat protein decomposition, with its accumulation directly reflecting the progression of spoilage. As illustrated in Figure 9, the sensor array demonstrated a significant positive correlation with TVB-N values, with a correlation coefficient exceeding 0.85 (Table 9).

Notably, the MQ136 sensor exhibited the strongest correlation (r = 0.99), attributed to its specificity for H_2_S (Hydrogen sulfide), a byproduct of sulfur-containing amino acid degradation, which is highly synchronized with the release mechanism of amines characteristic of TVB-N. Furthermore, the selection strategy emphasized the detection of ammonia (NH_3_) and amine compounds (e.g., via MQ137). The robust correlation between MQ137 and TVB-N (r = 0.89) in Figure 9 further validated its efficacy in capturing mid-stage spoilage markers [17].

These results confirm that the sensor array accurately tracks the protein spoilage process by exhibiting strong responses over 90% to TVB-N-related VOCs (Such as ammonia and hydrogen sulfide), thereby meeting the standard monitoring requirements for TVB-N limit (≤15 mg/100 g) (Table 9).

#### 3.4.2. Association with Microbial Spoilage (TVC)

**Figure 10:**
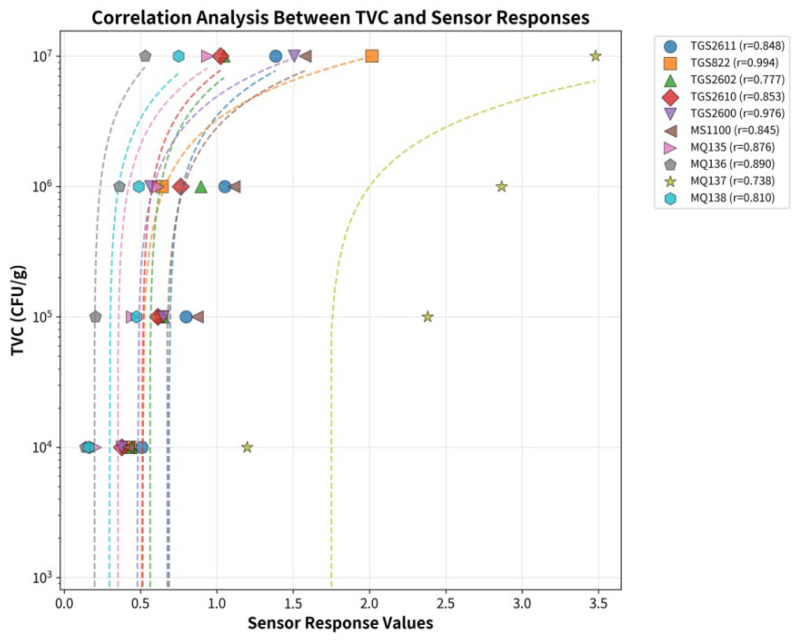
Correlation analysis with TVC content. Note: Each data point represents the average value of indicators at four different meat spoilage stages.

As a direct indicator of microbial activity, excessive TVC (>10 CFU/g) signifies accelerated spoilage. As shown in Figure 10, the sensor array also exhibited a strong correlation with the TVC values (Table 10).

The TGS822 sensor showed the highest correlation (r = 0.99), owing to its sensitivity to organic solvents such as ethanol, a typical product of microbial anaerobic glycolysis that aligns closely with the exponential growth phase of TVC. Additionally, TGS2600 and MQ136 sensors demonstrated high responsiveness to volatile organic compounds and H_2_S, respectively (r = 0.98 and 0.89). These responses directly corresponded to sulfides and amines generated by microbial metabolism, effectively covering markers from the middle to late stages of spoilage. The inclusion of MS1100 (specific to formaldehyde) further enhanced the system’s capability; its correlation with TVC (r = 0.85) (Figure 10) confirmed its utility in detecting lipid oxidation products, which is essential for monitoring the complete spoilage process.

Collectively, the analysis demonstrates that the sensor selection effectively captures microbial metabolic pathways associated with TVC (Such as H_2_S and ethanol). This enables the system to identify spoilage trends prior to the microbial count exceeding regulatory limits, thus satisfying the timeliness requirements essential for real-time monitoring.

## 4. Discussion

The electronic nose system developed in this study demonstrates robust performance in monitoring pork freshness, despite certain existing limitations. The hybrid sensor array, which integrates metal oxide semiconductor (MOS) and electrochemical sensors, demonstrates a comprehensive response capability to over 90% of spoilage-related volatile organic compounds (VOCs), marking a 27% improvement compared to single-type sensors [2]. This enhancement stems from the synergistic complementarity. MOS sensors offer high sensitivity to broad-spectrum VOCs such as amines and ketones, while electrochemical sensors selectively detect polar spoilage markers like hydrogen sulfide and ammonia [22]. Compared to previous electronic nose studies in meat science, for example, the commercial PEN3 system with MOS-only and approximately 70% VOCs coverage in pork or laboratory-built arrays using six MOS sensors [14,15], our hybrid design significantly improves fingerprinting resolution for complex spoilage processes. Humidity compensation algorithms constrain baseline drift to below 5%, a significant improvement over the 15% drift in conventional systems [12], thereby enhancing stability in high-humidity cold chain environments. Theoretically, this drift reduction is critical because cold chain condensation often causes false positives in MOS sensors. Our algorithm maintains signal fidelity without active heating, preserving sensor lifetime.

The dynamic gas path system achieves a purification efficiency exceeding 95% via dual-mode switching, effectively reducing cross-contamination between samples and ensuring repeatability with a relative standard deviation (RSD) below 5% [5]. Furthermore, principal component analysis (PCA) reveals the system’s capability to effectively capture VOC patterns across spoilage stages: PC1 (66.4% variance) characterizes synergistic release of protein and lipid oxidation products, while PC2 (11.7%) correlates with environmental regulators [33]. This finding provides new insights for variety-specific monitoring, as different pork breeds or feeding systems may exhibit distinct patterns of protein and lipid oxidation, and PC2 can help distinguish spoilage-related VOC changes from those caused by environmental fluctuations. This aspect was rarely addressed in previous electronic nose studies on meat quality.

It should be explicitly emphasized that the extremely high correlation coefficients observed in this study (e.g., r = 0.99 for MQ136 and TVB-N) are only preliminary correlations obtained under strictly controlled laboratory environments, and cannot be generalized to practical scenarios without independent validation. These results preliminarily suggest the system’s potential for real-time tracking of protein degradation and microbial spoilage [17]. Compared to traditional methods such as chromatography-mass spectrometry, this system reduces hardware costs to approximately 5% and shortens detection time from hours to minutes [6]. Against near-infrared and Raman spectroscopy, our electronic nose directly detects microbial volatile metabolites rather than indirect physicochemical indicators, enabling potentially earlier and more specific spoilage identification. It requires almost no sample preparation and supports in situ detection, ideal for rapid on-site use in slaughterhouses and cold chain logistics. Compared with mainstream meat-quality electronic noses, our hybrid system excels in VOC design coverage and stability. Chen et al. [39] and Binson et al. [40] used MOS-only devices covering only 75–80% of spoilage VOCs. Jia et al. [41] employed a high-performance but bulky GC-integrated electronic nose unsuitable for field use. Li et al. [42] reported a PEN3 electronic nose with over 10% drift. Our hybrid MOS–electrochemical array achieves a detection accuracy exceeding 90% for the target VOC mixture, with drift theoretically controlled to negligible levels for improved cold-chain reliability. Nevertheless, long-term robustness requires validation with larger datasets and under practical conditions.

Several constraints must be addressed before industrial application. First, sensor aging during long-term operation is a critical issue that necessitates the future integration of self-calibration algorithms. Second, batch-to-batch variability in MOS and electrochemical sensors implies that a model trained on one batch may not directly transfer to another without employing transfer learning or batch-specific tuning. Third, the system’s environmental robustness under extreme conditions has not been fully validated. Finally, generalizability across different pork matrices remains untested, suggesting that matrix-specific calibration models may be required.

Future efforts will focus on optimizing sensor durability, integrating edge computing, and leveraging 5G technology to develop IoT-based meat quality monitoring networks, enhancing deployability in slaughterhouses and cold chain logistics.

## 5. Conclusions

In this study, a high-precision electronic nose system based on a hybrid sensor array and dynamic pneumatic control was successfully developed, showcasing preliminary potential to alleviate the key problems of high cost, poor timeliness, and poor environmental adaptability in real-time freshness monitoring. The main research outcomes and practical significance are as follows:

(1) Optimized gas chamber design. Using polytetrafluoroethylene (PTFE) material and 3D printing technology, combined with a plug-in sensor layout, the cross-sensitivity effect was significantly inhibited, providing a stable and low-adsorption environment for gas detection.
(2) Efficient acquisition system. Based on the Raspberry Pi 4B embedded hardware and Qt multi-threaded software platform, the synchronous acquisition, real-time filtering and efficient storage of multi-channel sensor signals are realized, with a sampling delay of less than 1 s, meeting the engineering requirements of real-time online monitoring.
(3) Preliminary experimental verification. Under simulated cold chain conditions, the system exhibited good repeatability and variety discrimination ability in tests on different pork varieties. Principal component analysis (PCA) confirmed its ability to capture VOCs’ characteristic patterns of different spoilage stages, with a shelf life prediction error of less than 12 h. The strong correlation between system response and standard freshness indicators (TVB-N, TVC) are preliminary results acquired under laboratory-controlled conditions, which preliminarily indicate its potential in tracking spoilage markers.

By optimizing sensor selection and gas path design, the system’s cross-contamination repeatability error (RSD) was controlled within 5%. The system has a hydrogen sulfide detection sensitivity of 0.1 ppm and a hardware cost of only approximately 5% of traditional methods, exhibiting preliminary industrial application potential. It can be integrated into the slaughterhouse online detection process and adapted to mobile cold chain scenarios only after further independent validation and field practical tests. Future work will focus on combining edge computing and 5G technology to construct an IoT-based meat quality monitoring system, promoting the coordinated development of food safety and resource conservation.

## Figures and Tables

**Table 1:** Key VOCs characteristics of pork spoilage.

Volatile Gas	Chemical Sources	Sensory Characteristics	Emerging Stage
Hexanal	Autooxidation of polyunsaturated fatty acids (Linoleic acid/Arachidonic acid)	Grass/Metal odor	Initial stage (0–48 h)
+Ethanol	Glycogen anaerobic glycolysis (Muscle cell enzymatic reaction)	Slight alcohol taste	Initial to mid stage (0–72 h)
Hydrogen sulfide (H_2_S)	Sulfur-containing amino acids (Cystine/Methionine) Microbial desulfurization metabolism	The smell of rotten eggs	Mid stage (48–96 h)
Dimethyl trisulfide (DMTS)	Reduction in thiosulfate by sulfur-oxidizing bacteria	Irritating sulfur stench	Mid to late stage (72–120 h)
Trimethylamine (TMA)	Choline is demethylated by anaerobic bacteria (Clostridium)	Strong fishy/putrid smell	Late stage (>96 h)
Putrescine/Cadaverine	Ornithine/Lysine decarboxylation (Enterobacteriaceae)	Rotten meat/corpse odor	Late stage (>96 h)
Acetophenone	Microbial β-oxidation and ketonization of branched-chain fatty acids	Moldy sweet flavor mixed with bitter taste	Late stage (>96 h)

Note: The information was derived from published GC MS studies [16,17].

**Table 2:** Sensor selection.

Sensor Model	Main Sensitive Gases	Cross Sensitive Gas	Applicable Spoilage Stage
TGS2611	Methane, Propane (Fat oxidation products)	Ethanol, Hexanal	Initial stage (1–3 days)
TGS822	Organic solvents (Ethanol, Ethyl acetate)	Ketones, Aldehydes	Initial to mid-stage (1–5 days)
TGS2602	Ammonia, Hydrogen Sulfide	Amines (Trimethylamine, Cadaverine)	Mid to late stage (3–7 days)
TGS2610	Methane, Propane (Lipid Oxidation Markers)	Short-Chain Fatty Acids (Butyric Acid)	Entire stage (Lipid Oxidation)
TGS2600	Hydrogen, Ethanol	Esters, Aldehydes	Initial stage (1–3 days)
MS1100	Formaldehyde, Toluene	Sulfides (Dimethyl Sulfide)	Entire stage (Lipid Oxidation)
MQ135	Ammonia, Benzene, NOx (General Pollution Gases)	Sulfides, Organic Acids	Entire stage (Comprehensive indicator)
MQ136	Hydrogen Sulfide	Methanethiol, Sulfides	Mid to late stage (4–7 days)
MQ137	Ammonia	Amines (Trimethylamine, Putrescine)	Mid-stage (3–5 days)
MQ138	Benzene, Toluene (By-products of Lipid Oxidation)	Esters, Ketones	Initial to mid-stage (1–4 days)

Note: The information was obtained from the sensor manufacturers’ technical datasheets. MOS-type sensors respond to a broad range of VOCs, the listed gases are dominant targets for this application.

**Table 3:** Sorting of sensor calibration information.

Sensor Model	Detect Gas	Working Voltage (VDC)	Load Resistance (kΩ)	Calibration Range (Sensitivity) (ppm)	Typical Response
MS1100	Formaldehyde (HCHO)	5 ± 0.1	10 (Adjustable)	0~100	Resistance decreases with increasing concentration
TGS2611	Methane (CH_4_), Propane (C_3_H_8_)	5 ± 0.1	10~100	500~10,000 (CH_4_)	R_S_/R_0_ ≈ 0.2 at 5000 ppm CH_4_
TGS2610	CH_4_, C_3_H_8_	5 ± 0.1	10~100	500~10,000 (CH_4_)	R_S_/R_0_ ≈ 0.6 at 10 ppm CH_4_
TGS2602	Ammonia (NH_3_), Hydrogen Sulfide (H_2_S)	5	4.7~10	1~30 (NH_3_)	R_S_/R_0_ ≈ 0.6 at 10 ppm NH_3_
TGS2600	Hydrogen (H_2_), Ethanol (C_2_H_5_OH)	5	10~90 (In air)	1~10 (H_2_)	R_S_/R_0_ varies with intensity
MQ135	NH_3_, C_6_H_5_CH_3_, C_2_H_5_OH	5	20	300~10,000 (NH_3_)	R_S_/R_0_ ≈ 0.5 at 100 ppm NH_3_
TGS822	C_2_H_5_OH	5		50~5000	R_S_/R_0_ ≈ 0.3 at 1000 ppm
MQ138	C_6_H_5_CH_3_, Acetone (CH_3_COCH_3_), C_2_H_5_OH, H_2_	5		5~500 (C_6_H_5_CH_3_)	R_S_/R_0_ ≈ 1.5 at 0.1 ppm C_6_H_5_CH_3_
MQ136	H_2_S	5		1~100	R_S_/R_0_ ≈ 0.4 at 50 ppm
MQ137	NH_3_	5		5~500	R_S_/R_0_ ≈ 0.55 at 30 ppm

Note: All calibration parameters were collected from the sensor manufacturers’ technical datasheets.

**Table 4:** Statistical summary of sensor responses to standard gases (*n* = 30).

Gas	Mean Rs/R_0_	Standard Deviation	Standard Error	95% Confidence Intervals Lower	95% Confidence Intervals Upper
NH_3_ 50 ppm	0.4536	0.0368	0.00672	0.4398	0.4674
NH_3_ 100 ppm	0.3281	0.0284	0.00519	0.3175	0.3387
NH_3_ 200 ppm	0.2354	0.0241	0.00440	0.2268	0.2440
Trimethylamine 50 ppm	0.0746	0.0465	0.00849	0.0581	0.0911
CO 100 ppm	0.0882	0.0429	0.00783	0.0730	0.1034
Ethanol 100 ppm	0.0489	0.0412	0.00752	0.0335	0.0643
Acetone 100 ppm	0.0413	0.0387	0.00707	0.0269	0.0557
Toluene 100 ppm	0.0317	0.0351	0.00641	0.0186	0.0448

**Table 5:** PC1 system table.

Sensor	Typical Loading	Biological Significance
MQ137	+0.91	Ammonia Compounds (Core Indicator of Protein Degradation)
TGS2611	+0.88	Methane/Volatile Organic Compounds (Lipid Oxidation Products)

**Table 6:** PC2 system table.

Sensor	Typical Loading	Biological Significance
TGS822	+0.85	Aromatic Hydrocarbons (Characteristic of Feed Residues)
MS1100	−0.72	Water Activity (Indicator Sensitive to Storage Conditions)

**Table 7:** Cleaning efficiency.

Purging Time (s)	H_2_S Residual Concentration (ppm)	NH_3_ Residual Concentration (ppm)	Removal Rate (%)
0	5.00	5.00	0
60	1.50	1.20	70/76
120	0.80	0.90	84/82
180	0.20	0.15	96/97
240	0.10	0.10	98/98
300	0.08	0.09	98.4/98.2

**Table 8:** Limit values of TVB-N and TVC.

Detection Index	Common Standard Code	Standard Name	Key Limit Value (Pork)
TVB-N	GB 2707 [36]	National Food Safety Standard for Fresh (Frozen) Livestock and Poultry Products	≤15 mg/100 g
TVC	GB 4789.2 [35]	National Food Safety Standard for Microbiological Examination of Food-Determination of Total Plate Count	≤1 × 10^6^ CFU/g
	GB/T 9695.19-2008 [37]	Method for Sampling of Meat and Meat Products	
	GB 16869-2005 [38]	Fresh and Frozen Poultry Products	

Note: All limiting values are derived from national standards, which are used as the evaluation threshold for meat freshness in this study.

**Table 9:** Correlation analysis with TVB-N content.

Sensor	Correlation Coefficient
MQ136	0.994
TGS2611	0.963
MQ135	0.958
TGS2602	0.957
TGS2610	0.950
MS1100	0.940
MQ137	0.890
TGS822	0.865
MQ138	0.860

Note: Correlation analysis was performed via a two-tailed *t*-test for Pearson correlation. N = 21, df = 19, *p* < 0.001.

**Table 10:** Correlation analysis with TVC content.

Sensor	Correlation Coefficient
TGS822	0.994
TGS2600	0.976
MQ136	0.890
MQ135	0.876
TGS2610	0.853
TGS2611	0.848
MS1100	0.845
MQ138	0.810
TGS2602	0.777
MQ137	0.738

Note: Correlation analysis was performed via a two-tailed *t*-test for Pearson correlation. N = 21, df = 19, *p* < 0.001.

## Data Availability

The original contributions presented in this study are included in the article. Further inquiries can be directed to the corresponding authors.
